# *Helicobacter pylori* Infection and the Patterns of Gastric Mucin Expression in Children

**DOI:** 10.3390/jcm9124030

**Published:** 2020-12-13

**Authors:** Ana-Maria Teodora Domșa, Raluca Lupușoru, Dan Gheban, Alexandra Buruiană-Simic, Bogdan Alexandru Gheban, Camelia Lazăr, Cristina Maria Borzan

**Affiliations:** 1 Department of Pathology, “Iuliu Hațieganu” University of Medicine and Pharmacy, 400012 Cluj-Napoca, Romania; domsa_dora@yahoo.com (A.-M.T.D.); dgheban@gmail.com (D.G.); buruiana.alexandra@yahoo.com (A.B.-S.); ghebanbogdan@yahoo.com (B.A.G.); lazar.camelia@gmail.com (C.L.); 2 Department of Gastroenterology and Hepatology, “Victor Babes” University of Medicine and Pharmacy, 300041 Timisoara, Romania; 3 Department of Functional Sciences, “Victor Babes” University of Medicine and Pharmacy, 300041 Timisoara, Romania; 4 Department of Pathology, Emergency Clinical Hospital for Children, 400370 Cluj-Napoca, Romania; 5 Department of Public Health and Management, “Iuliu Hațieganu” University of Medicine and Pharmacy, 400012 Cluj-Napoca, Romania; borzancristina@yahoo.com

**Keywords:** *Helicobacter pylori*, gastritis, children, gastric mucins, immunohistochemistry

## Abstract

Background: The updated model for the mechanism of gastric carcinogenesis demonstrates that *Helicobacter pylori* (*H. pylori*) is a risk factor in every step of the process. The expression of certain gastric mucins is altered by *H. pylori* infection in adult patients. The aim of our research was to assess the impact of *H. pylori* infection on the expression of secretory mucins in the pediatric antral mucosa. Methods: Slides were stained with monoclonal antibodies for MUC5AC, MUC6 and MUC2, digitalized and scored using both a semiquantitative and a quantitative approach. Results: The expression of MUC5AC was significantly lower in infected children. Also, MUC2 expression was more pronounced in infected children. MUC6 expression did not differentiate between infected and noninfected children. Additionally, the presence of chronic inflammation significantly altered the expression of MUC6 and MUC2. The expression of MUC6 was significantly higher in patients with gastric atrophy. Conclusion: The minor differences in mucin expression at distinct ages might stem from different *H. pylori* exposure periods. Further research is needed to determine the particular patterns of expression according to age and to evaluate the effects of the interaction between *H. pylori* and mucins in the progression of the gastric carcinogenesis cascade.

## 1. Introduction

*Helicobacter pylori* is the etiological agent of the most common chronic infection in the world, both among adults and children [1]. The bacillus is also recognized as a class 1 carcinogen, the infection being considered the most important risk factor for gastric cancer [2]. The updated model for the mechanism of gastric carcinogenesis showed that *H. pylori* is not only present at the debut but it is an etiological factor in every step of the cascade [3].

The mucus layer that covers the gastric mucosa is comprised predominantly of mucins, high molecular weight glycoproteins which provide viscosity and protect the mucosa from bacterial invasion and the toxic effects of pepsin and hydrochloric acid [4].

The mucins are divided into two groups: membrane-bound and secreted. The human gastric mucosa expresses both membrane-bound (MUC1), and secreted mucins (MUC5AC and MUC6); MUC5AC is secreted by the superficial cells, and MUC6 is secreted in the gastric glands, both of them being expressed in the normal gastric mucosa [5]. Additionally, the fetal stomach can express MUC2 and MUC5B [6], mucins that are also found in the gastric mucosa of adults with premalignant lesions, such as the aberrant expression of MUC2 in patients with intestinal metaplasia [7].

Mucins have a specific ability to bind *H. pylori*, thus preventing the contact of the bacterium with the epithelial surface. A decrease in MUC5AC expression was observed in *H. pylori* positive patients compared to healthy patients; the low expression may facilitate the movement and epithelial attachment of the bacterium [8]. In addition, the expression of MUC6 is increased; considering the antibiotic effect of the mucin, this is likely a defense mechanism of the stomach against infection [4].

According to the Correa model, chronic inflammation triggers the process of carcinogenesis [9]. *H. pylori* infection is considered the major risk factor for gastric atrophy and intestinal metaplasia, the main precancerous lesions [10,11].

Gastric atrophy, defined as the loss of glands in the mucosa [12,13], is considered a precursor to intestinal metaplasia [11]. Intestinal metaplasia is defined as the replacement of the superficial, foveolar, and glandular epithelium of the gastric mucosa with intestinal-type epithelium [14]. It was categorized according to the expression of mucins, the most used classification being that of Jass and Filipe: type I, complete, positive for sialomucins; type II, incomplete, hybrid; type III, incomplete, positive for sulfomucins [15]. The complete type is similar to the epithelium of the small intestine and is positive for MUC2, secreted by goblet cells; the incomplete type is similar to the large intestine and positive for MUC5AC, MUC6 and MUC2 [16,17]. Unlike atrophy, intestinal metaplasia does not regress after *H. pylori* eradication [18,19], the treatment being capable only of slowing the progression of the lesions; patients with intestinal metaplasia have a 6-fold increased risk of developing gastric cancer [20].

The effects of *H. pylori* infection on mucin expression have been extensively studied in adults, but there is limited and conflicting viewpoints regarding pediatric patients [21,22]. Considering that pediatric *H. pylori* infection has particular clinical and paraclinical features when compared to adults, we aimed to analyze the effects of *H. pylori* infection on the patterns of gastric mucin expression in children.

## 2. Materials and Methods

A retrospective study was performed on 93 consecutive cases of pediatric patients who were submitted to the Emergency Clinical University Hospital for Children in Cluj-Napoca between 01.01.2017 and 31.12.2017 and underwent an upper gastrointestinal endoscopy (UGE) for the purpose of investigating various digestive symptoms; biopsy specimens were collected from the antrum, the body of the stomach and the duodenum. Patients who were older than 18 years as well as patients with a medical history of *H. pylori* infection were excluded from the study.

All the available slides originating from the antral mucosa were assessed according to the Updated Sydney System by the same experienced pathologist. To determine *H. pylori* status, the slides were stained with Hematoxylin and Eosin as well as Giemsa; the patient was labeled as “infected” if both stains were able to identify *H. pylori*.

Commercial ready-to-use monoclonal antibodies for MUC5AC, MUC6 and MUC2 (Leica Biosystems Inc, Buffalo Grove, IL, USA) were used to determine the expression of gastric mucins. Serial 4 µm sections from formalin-fixed and paraffin-embedded biopsies were stained using the Leica BOND MAX Automated Immunohistochemistry System (Leica Biosystems Inc, Buffalo Grove, IL, USA) according to the manufacturer’s specifications; at least three serial sections were processed for each stain for an antral biopsy. To prevent technical errors, staining of duodenal and gastric body tissue samples from the same patients were performed at the same time and were used as controls. We encountered one case of intestinal metaplasia that was excluded from the final analysis. We also excluded the cases with insufficient material for analysis. Following these two additional exclusion criteria we were left with 59 cases.

With the aid of a slide scanner (Pannoramic SCAN II, 3DHISTECH, Budapest, Hungary), the immunohistochemically stained glass slides of the antral biopsies were digitized at 20× objectives magnification.

Two other pathologists, who were unaware of the histopathological findings, independently analyzed the digital slides, using the Case Viewer software (3DHISTECH, Budapest, Hungary) for visualization.

One of them used a semiquantitative approach to score the immunohistochemically stained sections. For each of the three types of examined mucins, a grade was assigned for the percentage of positive cells (0—no positive cells, 1—< 10% positive cells, 2—10–50% positive cells, 3—51–80% positive cells, 4—> 80% positive cells) and one for the intensity of the staining (0—no staining, 1—mild staining, 2—moderate staining, 3—intense staining); finally, as described in previous studies, an immunoreactive score (IRS) was calculated as a product of multiplication between the two described grades, with values ranging from 0 to 12 [23,24].

**Figure 1:**
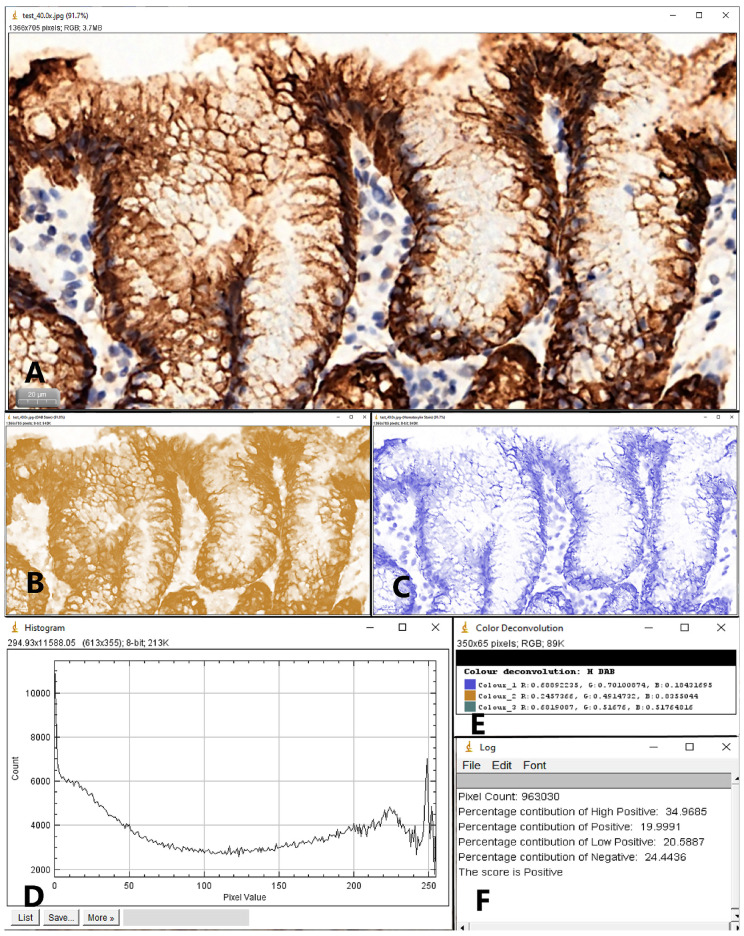
Example of color deconvolution for a MUC5AC stained slide (40× objective× magnification) using the IHC Profiler Plugin for ImageJ, in order to determine the Optical Density Score. (**A**) Sample image; (**B**) DAB stain; (**C**) Hematoxylin stain; (**D**) The histogram profile for the pixel intensity; (**E**) Color deconvolution values; (**F**) The intensity percentages used for the assignment of the score. ODS = (percentage of high positive ×4 + percentage of positive ×3 + percentage of low positive ×2 + percentage of negative ×1)/100.

The other pathologist used color deconvolution and pixel profiling for a quantitative analysis. For each of the three mucins, two images were captured at 40× objective magnification (performed with the Case Viewer software), one from the superficial region and one from the glandular region of the antral mucosa. The images were digitally processed with the IHC Profiler plugin for ImageJ (NIH, Bethesda, MD, USA). IHC Profiler is an open source plugin that analyses the pixel intensity and retrieves the percentage contribution of high positive, positive, low positive and negative pixels (Figure 1); using this percentages, an optical density score (ODS) was determined, for each of the two mucosal regions, according to a previously recommended formula [25,26]:

The study was approved by the ethical committee of the University of Medicine and Pharmacy Cluj-Napoca (208/16.05.2017) and was conducted in conformity with the Declaration of Helsinki. Prior to UGE, written informed consent was obtained from the parents or legal guardians of the patients. Demographic, clinical, and histological data were recorded anonymously.

The statistical analysis was conducted using MedCalc Statistical Software (MedCalc Software version 19.3.1, Ostend, Belgium) and Microsoft Office 2019.

Continuous data with normal distribution was depicted as mean ± SD. Nominal variables were presented as percentages. The Kolmogorov-Smirnov test was used to assess the normality of numerical variables. The differences between the groups were evaluated with the aid of parametric tests (student *t*-test and ANOVA) for continuous variables with normal distribution. For proportions, Fisher test and Pearson chi-squared test were applied. Linear regression was used for the appraisal of the association between numerical variables. Univariate and multivariate logistic regression models were used to test the influence of different dichotomous variables. The tests were considered significant at *p*-value < 0.05, with a 95% confidence level for intervals.

## 3. Results

The final analysis included 59 patients with a mean age of 13.3 ± 4.2 years; 40 were girls and 19 were boys. *H. pylori* positivity was detected in 29 (49.1%) patients. Regarding the *H. pylori* colonization grade, 19 (32.2%) were assessed as grade 1, 7 (11.8%) were assessed as grade 2 and 3 (5%) were assessed as grade 3 of colonization. Chronic inflammation was observed in 37 (62.7%) patients; in the *H. pylori* positive group 8 patients presented mild chronic inflammatory infiltrate, 11 patients presented moderate inflammation and 10 patients presented severe inflammation, while in the *H. pylori* negative group 8 patients presented mild inflammation and the rest of 22 patients presented no inflammatory infiltrate. Atrophy was identified in 15 (25.4%) cases.

**Figure 2:**
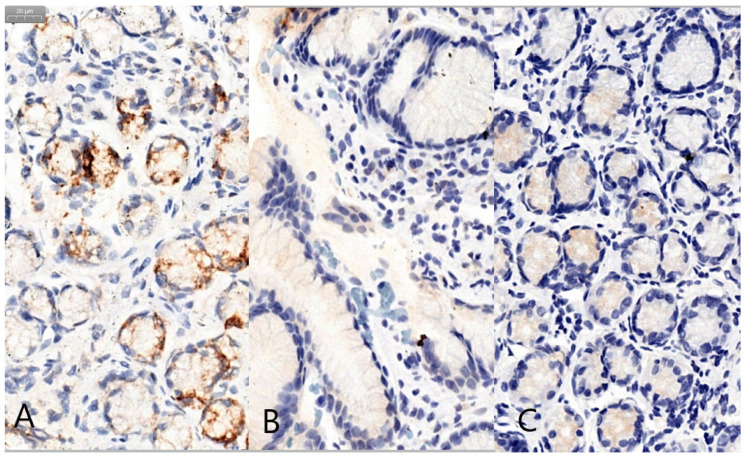
Examples of aberrant mucin expression (40× objectiveX): glandular expression of MUC5AC (**A**), foveolar/superficial expression of MUC6 (**B**) and glandular expression of MUC2 in the gastric epithelium (**C**).

Following the semiquantitative evaluation based on a visual scale, we observed an aberrant glandular expression of MUC5AC in 2/29 (6.9%) patients in the *H. pylori* positive group and in 2/30 (6.7%) patients in the *H. pylori* negative group, without significant difference between groups (*p* = 0.97). We encountered an aberrant expression of MUC6 in the foveolar/superficial epithelium in 1/29 (3.5%) patients in the *H. pylori* positive group and 4/30 (13.4%) in the negative group, with no statistical significance (*p* = 0.17). We also observed an aberrant expression of MUC2 in 2/29 (6.9%) patients in the *H. pylori* positive group and in 2/30 (6.7%) patients in the *H. pylori* negative group (Figure 2).

Using the two previously described methods for the evaluation of the immunohistochemical stains via the determination of the aforementioned IRS and ODS scores, we compared the expression of secretory mucins between the two groups (with *H. pylori* and without *H. pylori*). Table 1 illustrates the mean values of the two determined scores for the three analyzed mucins in different areas of the antral mucosa. We found significant differences between *H. pylori* positive and *H. pylori* negative pediatric patients in the expression of MUC5AC and MUC6, both in the superficial/foveolar region of the mucosa (Table 1).

**Figure 3:**
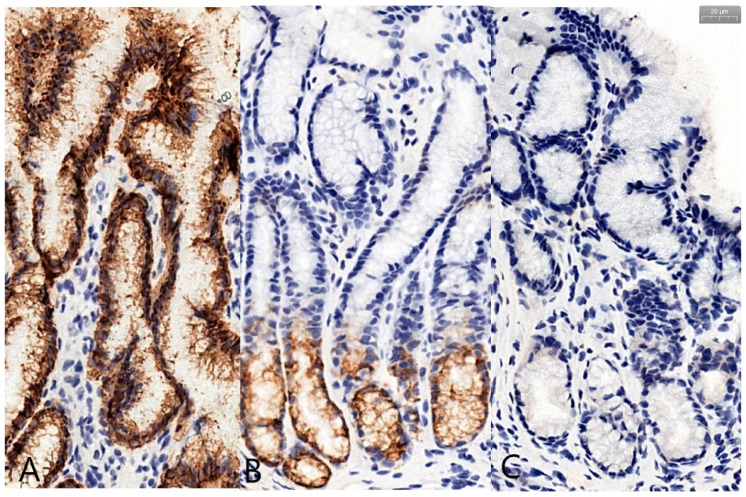
Examples of mucin expression (40× objectiveX): MUC5AC scored as IRS = 12 (**A**), MUC6 scored as IRS = 8 (**B**) and MUC2 scored as IRS = 0 (**C**).

Figure 3 presents representative images for the expression of the evaluated mucins, with IRS scores close to the averages of the two studied groups.

Regarding inflammation (Table 2), we found significant statistical differences between the group without inflammation and the group presenting chronic inflammation in the expression of MUC6, both in the superficial/foveolar region (*p* = 0.03) and in the glandular region of the mucosa (*p* = 0.02). The ODS score was also significantly different for the expression of MUC2 in the glandular compartment of the mucosa (*p* = 0.01).

Considering atrophy (Table 3), the IRS score was significantly higher for the expression of MUC6 (*p* = 0.02); furthermore, the ODS score was significantly increased for the expression of MUC6 in the glandular compartment for cases that showed atrophy compared to those without atrophic changes (*p* = 0.007).

Investigating the association between MUC5AC and MUC6 expression in *H. pylori* positive cases, by comparing the IRS scores, we observed that r = 0.36, R² = 0.12, *p* = 0.004, meaning that 12% of the IRS values for MUC5AC could be explained by the IRS values for MUC6. Furthermore, when comparing the ODS scores for the expression of MUC5AC in the superficial/foveolar epithelium with the expression of MUC6 in the glandular epithelium we observed that r = 0.31, R² = 0.09, *p* = 0.01, meaning that 9% of the ODS values for MUC5AC could be explained by the ODS values for MUC6.

We compared the two methods by which we evaluated the immunohistochemical expression of mucins and we found a weak correlation between the IRS score and the ODS score determined in the superficial/foveolar region of the antral mucosa for the expression of MUC5AC (r = 0.20, *p* < 0.001), and also between the IRS score and the ODS score determined in the glandular region of the antral mucosa for the expression of MUC6 (r = 0.01, *p* = 0.93).

When analyzing the influence of *H. pylori* infection on the ODS scores, we found that *H. pylori* positivity is increasing the ODS score for MUC5AC expression in the superficial/foveolar region by 2.09 times, OR = 2.09, 95%CI (0.25–17.7), *p* = 0.87 and the ODS score for MUC6 expression in the superficial/foveolar region by 9.5 times, OR = 9.5, 95% CI (0.87–103.61), *p* = 0.78; the ODS score for MUC5AC expression in the glandular region had an OR = 1.01, 95% CI (0.08–12.66), *p* = 0.89; the ODS score for MUC6 expression in the glandular region had an OR = 1.26, 95% CI (0.18–8.92), *p* = 0.41; the ODS score for MUC2 expression in the superficial/foveolar region had an OR = 3.86, 95% CI (0.36–40.9), *p* = 0.002 and the ODS score for MUC2 expression in the glandular region had an OR = 0.91 95% CI (0.19–4.28), *p* = 0.01.

## 4. Discussion

The aim of this study was to evaluate the influence of *H. pylori* infection on the expression of secretory mucins in the antral mucosa of pediatric patients. Current literature states that persistent *H. pylori* infection causes chronic inflammation, consecutive atrophy of the gastric mucosa, followed by intestinal metaplasia and the gastric carcinogenesis cascade extends over several years, the outcome of the disease being dependent on the age of acquisition of the infection [27,28]. Also, the expression of mucins influences the mode of *H. pylori* adhesion and consequently the persistence of the infection in the gastric mucosa [8]. Therefore, the identification of particular patterns of mucin expression in children could help to identify patients who would have a higher risk of developing gastric cancer, with a faster evolution along the steps of the carcinogenesis cascade and thus providing a basis for a closer follow-up of these patients.

To date, numerous studies showed that there is a dynamic interaction between the bacterium and the host; mucins have the potential to be active determinants on the disease outcome in *H. pylori* infection, their presence affecting proliferation, gene expression and *H. pylori* virulence [29]. Furthermore, the binding of *H. pylori* is influenced both by mucin glycosylation and by the adhesins expressed by the bacteria [30].

It was established that MUC5 and MUC6 are the major secretory mucins in the human stomach [31]. Usually, MUC5 and MUC6 are intensely expressed in the gastric mucosa of adults [5]. When analyzing the values of the IRS score for MUC5AC and MUC6, we observed that these were higher for MUC5AC, both in *H. pylori* negative and in *H. pylori* positive patients, meaning that the extent of the expression is lower for MUC6. This observation is supported by a previous study involving children, where MUC6 had a weak expression in patients under 15 years [32].

Pediatric studies report different patterns of expression in *H. pylori* positive patients, ranging from the absence of the aberrant expression of MUC6 and MUC2 [22], to an aberrant nuclear expression of MUC6 and MUC2 in a limited number of cases [32]. Meanwhile, research involving infected adults reports minimal expression of MUC5 in the deep glandular region [33] and an aberrant expression of MUC6 in the surface epithelium [34,35], going as far as 72% of infected adults; these differences may be explained by a longer *H. pylori* exposure in adult patients [34]. Although not significantly different between the infected and noninfected groups, we observed glandular expression of MUC5AC, foveolar expression of MUC6 and aberrant expression of MUC2 in the gastric epithelium in a limited number of cases. These aberrant expressions, both nuclear as well as the unusual topographic location, should be interpreted with caution, taking into account that the usual expression of mucins is at the cytoplasmic level of the epithelial cells, with a specific topographic distribution within the regions of the gastric mucosa [31].

As concluded by a meta-analysis that included 11 case-control studies, comparing human gastric mucin expression using immunohistochemistry in *H.pylori* positive and negative patients, the infection causes decreased MUC5AC expression and increased MUC6 expression [4].

MUC5AC is considered the main protective factor of the gastric mucosa [36]. *H. pylori* colocalizes with MUC5AC in the process of colonization [37]; the blood group antigen binding adhesin (BabA), an outer membrane protein produced by *H. pylori*, binds to the Lewis^b^ blood group antigen of the host, expressed by MUC5AC producing epithelial cells, this being the dominant path of adhesion [8,38,39]. It has been shown that *H. pylori* infection decreases the expression of MUC5AC [34,35]. Moreover, experiments conducted on murine gastric mucosa have demonstrated a decreased rate in mucin turnover in gastric surface epithelial cells in *H. pylori* infected mice compared to non-infected mice, indicating that by decreasing the mucosal defense mechanisms that remove bacteria through the mucus flow, *H. pylori* tries to create itself a suitable environment [40]. Another interesting observation made by an experimental study was that MUC5AC production is up-regulated in the acute, early phase of the infection, and this could promote the gastric colonization by *H. pylori* [41].

A similar expression of MUC5AC to that described in adults is also found in pediatric patients [22,32]. Our results confirm the data available in the literature, the ODS score for the expression of MUC5AC in the superficial/foveolar epithelium being decreased in infected patients when compared to noninfected patients.

Among studies examining adult patients, it has been established that MUC6 expression is increased in *H. pylori* infection [4], with the potential to lower the degree of colonization through its antibiotic properties [42]. It has been also shown that the up-regulation associated with *H. pylori* infection is reversible, the levels of MUC6 decreasing after eradication [43]. In children, one study reports that MUC6 expression is not significantly different in *H. pylori* infected patients from those uninfected [32]. Similarly, in our study, the IRS and the ODS score evaluating the extent of MUC6 expression in the glandular epithelium did not differentiate between *H. pylori* positive and *H. pylori* negative patients.

The expression of mucins might also be affected by the presence of inflammation; a complementary shift in mucin expression was described depending on the degree of inflammation and the bacterial colonization grade, MUC5AC expression being negatively affected, while MUC6 expression was positively affected [44]. In children, a decrease in MUC5AC expression has been described [30], probably in relation to the duration of the chronic inflammation [22,45], while in adults the expression of MUC5AC was influenced by the presence of active inflammation [44]. Our results contrast with previous studies, MUC5AC expression remaining unchanged in relation to inflammation, both when evaluated by the IRS and by the ODS scores (*p* > 0.05).

Regarding MUC6, the ODS scores for the expression in both the superficial and the glandular epithelium were significantly increased by the presence of chronic inflammation (*p* < 0.05). Also, the ODS score for the expression of MUC2 in the glandular region was significantly increased (*p* = 0.01); the ODS score for the expression of MUC2 in the superficial region of the mucosa was higher for the patients presenting chronic inflammation, but without achieving statistical significance. In adults, the expression of MUC6 is positively influenced by the presence of inflammation [44]; no previous significant data was found for the relationship between MUC6 and MUC2 expression and inflammation in children.

Gastric atrophy, the step that precedes metaplasia in the gastric carcinogenesis cascade, and intestinal metaplasia are rare findings among children infected with *H. pylori*, probably due to the limited time of exposure to the bacterium [46,47]. Intestinal metaplasia is one of the lesions that paves the way for the development of gastric carcinoma and according to previous studies, a distinct pattern of mucus expression associated with this precursor lesion was determined through histochemical and immunohistochemical techniques: the incomplete form is defined by coexpression of MUC5AC and MUC6 together with MUC2, while the complete form is associated with a strong expression of MUC2 along with little or no expression of MUC5AC and MUC6 [48].

In adults, investigators agree on a decrease in the expression of gastric mucins MUC6 and MUC5AC and de novo expression of MUC2, an intestinal-type of mucin [49]. Moreover, MUC5AC gradually decreases along with the progression of the types of metaplasia [50]. Studies conducted on gastric cancer cells showed that MUC5AC expression is significantly lower in gastric cancer than in *H. pylori* negative patients [51,52,53] and is higher in the well differentiated forms than in less differentiated forms [51]. Furthermore, the expression of MUC5AC decreases along with the local invasion of the tumor and with the increase in the number of lymph nodes with metastasis [54]. Apparently, MUC5AC has a prognostic value for gastric cancer patients, being positively associated with the disease-free survival period [55]. Regarding MUC6, a study that evaluated the types of gastric epithelial neoplasia showed that MUC6 expression is associated with high-grade dysplasia and intramucosal cancer, the expression of the mucin being associated with an increased risk of malignant transformation [56]. Previous research evaluating genetic variability in mucin genes of patients presenting gastric cancer precursor lesions has also shown that the genetic variability of MUC2 influences the progression of the lesions in *H. pylori* infected patients [57].

Among studies that included pediatric patients, the expression of MUC5AC, MUC6 and MUC2 were all increased in patients with intestinal metaplasia, but no data was available for gastric atrophy [32]. In our study, both the IRS and ODS scores for MUC6 expression in the glandular epithelium were significantly higher in patients with gastric atrophy. The ODS and IRS scores for MUC5AC and MUC2 expression did not differ significantly, but we observed an upward trend of expression for cases with atrophy compared to those without atrophy.

An interesting finding of this study in regard to the expression of MUC2, was the trend toward an increased expression in *H. pylori* infected patients, for all the three determined scores, even if without reaching statistical significance. Also, the odds ratio for the ODS scores that evaluate MUC2 expression, both in the foveolar and in the glandular epithelium, indicate a more pronounced expression in cases that are *H. pylori* positive.

A fair agreement between the semi-quantitative approach based on a visual scale and the quantitative analysis based on color deconvolution was observed. Some of the factors that could possibly explain these results are related to the determination of the IRS scores based on the subjective but experienced analysis of the pathologist and to the automatic determination of the ODS scores. The latter could be more sensitive to small differences in colorability, but on the other hand could miss the slight technical variations of immunohistochemical staining; it would be necessary to deepen research on a larger number of patients to see if these differences persist. Even if our study detected a fair agreement between the IRS and ODS scores, it can be observed that the increasing or decreasing trends are similar between the two scores.

To the best of our knowledge, there are only two other studies that evaluate the expression of these three mucins (MUC5AC, MUC6 and MUC2) in the gastric mucosa of children, with a mean age of 12.2 years and a range from 3 to 18 for the first study [22]. In the second study the age of the patients ranged from 1.4 to 35 years, of the patients included none being between the ages of 15 and 19 [32]. One strength of our study is the fact that the cohort’s mean age was 13.3 years, the oldest patient being 18. Another important aspect is that previous research, both in adults and children, used semi-quantitative techniques to score the staining extent, while we evaluated the staining intensities by two different methods, both quantitative (through color deconvolution and the ODS scores) and semi-quantitative (using visual scales to determine the IRS scores).

The limitations of our study are related to the retrospective nature of the research and to the limited number of pediatric patients, which prevented the division into subgroups according to age and the bacterial colonization grade. Another limitation is related to the determination of the ODS scores. For the design of the study, we considered the recommendations of the IHC profiler developers; they describe the impact of magnification on image scoring and state that a 40x magnification is more appropriate in achieving correct pathological scoring and that increasing the magnification allows focusing on the element of interest and minimizes the effect of the stromal component [26]. To overcome these limitations, prospective large-scale studies are needed with the evaluation of several immunohistochemically stained sections for the determination of the ODS scores that would increase the power of the statistical analysis; these could bring significant benefits in establishing the potential associations between *H. pylori* infection, the type and severity of the inflammatory response, the different progressive steps of the gastric carcinogenesis cascade and the expression of mucins.

## 5. Conclusions

In conclusion, our study supports to some extent the data available from previous pediatric research but also indicates that there are slight differences between mucin expression at different ages. Inconsistency of mucin expression might stem from the different duration of exposure to *H. pylori* infection.

Given the variability of mucin expression in the literature and considering that the majority of the studies are based on semi-quantitative scoring methods, thus influenced by the subjectivity of the examiner, future work is needed to establish the particular patterns of mucin expression, both in adults and children and to evaluate if the association between *H. pylori* infection and mucin expression plays a role in the progression of the gastric carcinogenesis cascade. More precisely, mucins could act as prognostic factors for patients at high risk of developing gastric cancer.

A special attention should be paid to the expression MUC2, which may increase even before the pathologist is able to determine on routine stains that patients have intestinal metaplasia; establishing a relationship between the extent of expression and age groups has the potential to determine the onset of premalignant lesions.

## Figures and Tables

**Table 1:** Comparison of mucin expression in the antral mucosa between *H. pylori*+ and *H. pylori*− groups.

Type of Mucin	Determined Score	*H. pylori*+	*H. pylori*−	*p*-Value
MUC5AC	IRS	11.21 ± 1.55	10.81 ± 1.90	0.41
MUC6	IRS	8.55 ± 3.11	9.21 ± 3.01	0.37
MUC2	IRS	0.31 ± 0.24	0.21 ± 0.71	0.72
MUC5AC	ODS in superficial/foveolar region	2.28 ± 0.23	2.33 ± 0.24	0.04
MUC5AC	ODS in glandular region	1.65 ± 0.23	1.64 ± 0.17	0.88
MUC6	ODS in superficial/foveolar region	1.63 ± 0.24	1.76 ± 0.22	0.04
MUC6	ODS in glandular region	2.05 ±0.08	2.09 ± 0.23	0.58
MUC2	ODS in superficial/foveolar region	1.73 ± 0.20	1.66 ± 0.24	0.26
MUC2	ODS in glandular region	1.68 ± 0.21	1.61 ± 0.11	0.23

Variables are presented as mean ± standard deviation; *H. pylori*+ = *Helicobacter pylori* positive; *H. pylori*− = *Helicobacter pylori* negative; IRS = immunoreactive score; ODS = optical density score.

**Table 2:** Comparison of mucin expression in the antral mucosa between cases presenting chronic inflammation and cases without inflammation.

Type of Mucin	Determined Score	Inflammation+	Inflammation−	*p*-Value
MUC5AC	IRS	11.18 ± 1.57	10.81 ± 2.01	0.43
MUC6	IRS	8.86 ± 3.11	9.00 ± 3.35	0.87
MUC2	IRS	0.32 ± 0.17	0.13 ± 0.63	0.49
MUC5AC	ODS in superficial/foveolar region	2.31 ± 0.22	2.30 ± 0.28	0.86
MUC5AC	ODS in glandular region	1.65 ± 0.21	1.63 ± 0.17	0.71
MUC6	ODS in superficial/foveolar region	1.78 ± 0.18	1.67 ± 0.27	0.03
MUC6	ODS in glandular region	2.13 ± 0.24	1.97 ± 0.27	0.02
MUC2	ODS in superficial/foveolar region	1.73 ± 0.24	1.64 ± 0.19	0.13
MUC2	ODS in glandular region	1.70 ± 0.25	1.55 ± 0.19	0.01

Variables are presented as mean ± standard deviation; Inflammation+ = presence of chronic inflammation; Inflammation− = without chronic inflammation; IRS = immunoreactive score; ODS = optical density score.

**Table 3:** Comparison of mucin expression in the antral mucosa between cases presenting atrophy and cases without atrophy.

Type of Mucin	Determined Score	Atrophy+	Atrophy−	*p*-Value
MUC5AC	IRS	11.46 ± 1.40	10.90 ± 1.84	0.28
MUC6	IRS	10.46 ± 2.06	8.38 ± 3.22	0.02
MUC2	IRS	0.60 ± 0.68	0.13 ± 0.63	0.12
MUC5AC	ODS in superficial/foveolar region	2.30 ± 0.24	2.30 ± 0.25	0.95
MUC5AC	ODS in glandular region	1.60 ± 0.15	1.66 ± 0.21	0.32
MUC6	ODS in superficial/foveolar region	1.78 ± 0.12	1.67 ± 0.27	0.13
MUC6	ODS in glandular region	2.22 ±0.21	2.02 ± 0.26	0.007
MUC2	ODS in superficial/foveolar region	1.74 ± 0.23	1.68 ± 0.22	0.37
MUC2	ODS in glandular region	1.72 ± 0.19	1.62 ± 0.25	0.12

Variables are presented as mean ± standard deviation; Atrophy+ = presence of atrophy; Atrophy− = absence of atrophy; IRS = immunoreactive score; ODS = optical density score.
